# Fast Cerebellar Reflex Circuitry Requires Synaptic Vesicle Priming by Munc13-3

**DOI:** 10.1007/s12311-015-0645-0

**Published:** 2015-01-24

**Authors:** Pallavi Rao Netrakanti, Benjamin H. Cooper, Ekrem Dere, Giulia Poggi, Daniela Winkler, Nils Brose, Hannelore Ehrenreich

**Affiliations:** 1 Clinical Neuroscience, Max Planck Institute of Experimental Medicine, Göttingen, Germany; 2 Department of Molecular Neurobiology, Max Planck Institute of Experimental Medicine, Göttingen, Germany; 3 DFG Research Center for Nanoscale Microscopy and Molecular Physiology of the Brain, Göttingen, Germany

**Keywords:** Hippocampus, Immunohistochemistry, Acoustic startle response, IntelliCage, Spatial working and reference memory, Gender

## Abstract

Munc13-3 is a member of the Munc13 family of synaptic vesicle priming proteins and mainly expressed in cerebellar neurons. *Munc13*-*3* null mutant (*Munc13*-*3* ^−/−^) mice show decreased synaptic release probability at parallel fiber to Purkinje cell, granule cell to Golgi cell, and granule cell to basket cell synapses and exhibit a motor learning deficit at highest rotarod speeds. Since we detected Munc13-3 immunoreactivity in the dentate gyrus, as reported here for the first time, and current studies indicated a crucial role for the cerebellum in hippocampus-dependent spatial memory, we systematically investigated *Munc13*-*3* ^−/−^ mice versus wild-type littermates of both genders with respect to hippocampus-related cognition and a range of basic behaviors, including tests for anxiety, sensory functions, motor performance and balance, sensorimotor gating, social interaction and competence, and repetitive and compulsive behaviors. Neither basic behavior nor hippocampus-dependent cognitive performance, evaluated by Morris water maze, hole board working and reference memory, IntelliCage-based place learning including multiple reversals, and fear conditioning, showed any difference between genotypes. However, consistent with a disturbed cerebellar reflex circuitry, a reliable reduction in the acoustic startle response in both male and female *Munc13*-*3* ^−/−^ mice was found. To conclude, complete deletion of *Munc13*-*3* leads to a robust decrease in the acoustic startle response. This readout of a fast cerebellar reflex circuitry obviously requires synaptic vesicle priming by Munc13-3 for full functionality, in contrast to other behavioral or cognitive features, where a nearly perfect compensation of Munc13-3 deficiency by related synaptic proteins has to be assumed.

## Introduction

In the past decade, studies in animals and man have implicated the cerebellum in the processing of signals that are essential not only for motor function but also for perception, cognition, and emotion [1–3]. Spatial cognition, for instance, involves parallel information processing, relational memory, and context-dependent action selection [4–7]. In this framework, the cerebellum is crucial for procedural spatial learning [8–10] and the formation of a hippocampal spatial code [11]. The formation of a hippocampal spatial code [11] or a spatial cognitive map [12], which are used by mammals for spatial orientation and navigation in space, depends not only on environmental cues but also on self-motion signals that are relayed to the hippocampus by the cerebellum [13].

Mammalian Unc-13 (Munc13) proteins constitute a family of molecules (Munc13-1, Munc13-2, Munc13-3, Munc13-4, and Baiap3) with homology to *Caenorhabditis elegans* Unc-13 [14]. Among these, Munc13-1, Munc13-2, and Munc13-3 are largely brain-specific. Their function is to prime secretory vesicles for Ca^2+^-triggered exocytosis in neurons and neuroendocrine cells. Munc13 proteins contain a conserved C-terminal domain that promotes the assembly of SNARE complexes for vesicle priming [15]. Munc13-1 is expressed in all regions of the rodent CNS [16], whereas Munc13-2 and Munc13-3 exhibit strikingly different expression patterns. Munc13-2 is only present in rostral brain regions, including cerebral cortex and CA regions of the hippocampus, whereas Munc13-3 is almost exclusively expressed in the cerebellum, most strongly in cerebellar granule cells that target the protein to their presynaptic parallel fiber axon terminals, and in Purkinje cells [17].

Studies on *Munc13*-*3* null mutant (*Munc13*-*3* ^−/−^) mice, which show normal cerebellar morphology and cytoarchitecture [17], uncovered a widespread but subtle role of Munc13-3 in setting the release probability of multiple cerebellar synapse types, which in turn affects cerebellar synaptic transmission and short-term plasticity. Synaptic transmission at granule cell to Purkinje cell synapses of *Munc13*-*3* ^−/−^ mice is impaired due to a decrease in presynaptic transmitter release probability, which likely results from a subtle synaptic vesicle priming defect [17]. The reduced release probability at granule cell to Purkinje cell synapses in *Munc13*-*3* ^−/−^ mice becomes manifest as altered short-term plasticity. It is characterized by subtly but significantly increased paired-pulse facilitation [17], a very slight, non-significant increase in steady-state facilitation during 14 Hz action potential trains [17], and significantly increased synaptic facilitation upon stimulation at 50 Hz [18]. Granule cell to Golgi cell synapses in *Munc13*-*3* ^−/−^ mice show a slightly more aggravated phenotype than granule cell to Purkinje cell synapses, characterized by a stronger increase in paired-pulse facilitation and by a substantial increase in synaptic facilitation during 50 Hz trains of ten action potentials [19]. However, a longer-lasting type of short-term plasticity, post-tetanic potentiation, is not changed at these synapses in *Munc13*-*3* ^−/−^ mice [19].

Wild-type granule cell to basket cell and granule cell to stellate cell synapses in the cerebellum show strikingly different short-term plasticity features, and the two synapse types exhibit differential Munc13-3 dependence [18]. Whereas granule cell to basket cell synapses exhibit transient and only very subtle paired-pulse facilitation, followed by a pronounced depression at 50 Hz stimulation frequencies, granule cell to stellate cell synapses show strong facilitation under the same stimulation conditions, similar to synapses between granule cells and Purkinje cells [18]. In *Munc13*-*3* ^−/−^ mice, paired-pulse facilitation is strongly increased in granule cell to basket cell synapses whereas granule cell to stellate cell synapses are not affected by Munc13-3 loss [18]. Interestingly, loss of Munc13-3 does not directly affect inhibitory synaptic transmission exerted by stellate or basket cells. However, because the deletion of Munc13-3 renders granule cell to basket cell synapses more facilitating, the tonic inhibition that is exerted upon Purkinje cells by the disynaptic connection from granule cells via basket cells to Purkinje cells during 50 Hz stimulation trains is increased in *Munc13*-*3* ^−/−^ mice [18].

Changes in long-term plasticity as a consequence of Munc13-3 loss have not been assessed in any of the synaptic connections studied so far [17–19]. In fact, such changes are somewhat improbable to occur in *Munc13*-*3* ^−/−^ cerebellum, given the rather subtle short-term plasticity changes in these mice, which are unlikely to affect the induction, expression, or maintenance of long-term plasticity. This notion is supported by the fact that the loss of Munc13-2 in hippocampal mossy fiber synapses leads to changes in short-term plasticity that are very similar to the synaptic changes detected in the cerebellum of *Munc13*-*3* ^−/−^ mice but does not affect long-term potentiation at these synapses [20].

In spite of the fact that Munc13-3 deficiency affects cerebellar neurotransmission in a multifaceted way, the behavioral consequences of Munc13-3 loss that have been determined so far are rather minor. *Munc13*-*3* ^−/−^ mice do not show any overt changes that would indicate a major cerebellar dysfunction, such as ataxia or motor coordination defects. The only phenotypic change that has been detected in *Munc13*-*3* ^−/−^ mice so far is a motor learning deficit at very high rotarod speeds [17].

Taking advantage of new antibodies to Munc13-3, we found recently that Munc13-3 is present not only in cerebellar synapses but also in perforant path terminals targeting the dendrites of granule cells in the hippocampus, as exemplified in the present study. Functional changes in synaptic transmission within the intact hippocampus have so far only been studied in *Munc13*-*2* ^−/−^ mice [20], mainly because loss of Munc13-1 causes an almost total arrest of synaptic transmission in hippocampal neurons and perinatal death [21] and because the low levels of Munc13-3 in hippocampus had not been detectable until very recently, which made corresponding analyses of hippocampal synaptic transmission in *Munc13*-*3* ^−/−^ mice seem superfluous. As stated above, loss of Munc13-2 in the hippocampus alters mossy fiber synaptic transmission in the CA3 region, but it leaves Schaffer collateral synapses, associational-commissural synapses, and inhibitory synapses targeting CA3 pyramidal neurons unaffected. Specifically, mossy fiber synapses of *Munc13*-*2* ^−/−^ mice show reduced synaptic strength as reflected by the ratio between fiber volley and fEPSP amplitudes and an increased failure rate as assessed by evoked excitatory postsynaptic currents (EPSCs), reduced transmitter release probability as assessed by the sensitivity of evoked EPSC to the low-affinity AMPA receptor antagonist γ-DGG, and increased paired-pulse and frequency facilitation, whereas long-term potentiation remains normal [20].

The present study was designed to perform for the first time a comprehensive behavioral phenotyping of *Munc13*-*3* ^−/−^ mice of both genders, assessing cerebellum-dependent behavior and including an extensive cognitive test battery focusing on hippocampus-related performance.

## Materials and Methods

### Generation and Genotyping of Munc13-3 Null Mutant Mice

The generation and genotyping of male and female *Munc13*-*3* ^−/−^ mice have been described elsewhere [17]. The mice used in this study were generated via homologous recombination in embryonic stem cells derived from 129SV mice and thus had initially a mixed 129SVxC57BL/6J genetic background. Therefore, the genetic background was homogenized by backcrossing to the C57BL/6J strain for more than ten generations. Details on the generation and characteristics of Munc13-3-enhanced green fluorescent protein (EGFP) knock-in mice were published previously [22].

### Immunohistochemistry

Wild-type and Munc13-3-EGFP mice were sacrificed at 8 and 14 weeks of age by decapitation upon isoflurane anesthesia. Brains were removed and rapidly frozen in isopentane cooled to −35 °C. Cryosections (20 μm) were made through the dorsal hippocampus (coronal plane) and the cerebellum (sagittal plane) and thaw-mounted on Superfrost slides. To ensure comparable conditions for fixation and immunolabeling, sections from both genotypes were collected on each slide and fixed by immersion in methanol at −20 °C for 10 min. Sections were incubated for 90 min at room temperature in blocking solution (0.1 M phosphate buffer (PB), 5 % normal goat serum, 0.3 % Triton X-100, pH 7.4) and then overnight at 4 °C with primary antibodies (mAb rabbit anti-GFP, Invitrogen G10362, dilution 1:20; pAb guinea pig anti-VGLUT1, Synaptic Systems, dilution 1:1000), diluted in incubation buffer (0.1 M PB, 3 % normal goat serum, 0.1 % Triton X-100, pH 7.4). Slides were washed extensively in PB and then incubated 2 h at room temperature in the dark with fluorescent secondary antibodies (Alexa 488-coupled goat anti-rabbit and Alexa 555-coupled goat anti-guinea pig, Invitrogen, 1:2000) diluted in incubation buffer. Coverslips were mounted on slides with Aqua-PolyMount (Polysciences, Warrington, PA). Low-magnification overviews revealing the distribution of Munc13-3-EGFP signal in the hippocampus and cerebellum were acquired with a Leica MZ16F fluorescent stereomicroscope, equipped with a 1.4-MP digital camera (Leica DFC 350 FX). Wild-type and Munc13-3-EGFP sections were imaged using identical imaging parameters. High-magnification confocal laser scanning micrographs (512 × 512; pixel spacing *x*, *y* = 75.8 nm) of Munc13-3-EGFP and VGLUT1 signals in selected regions of the dentate gyrus and cerebellum were acquired in sequential scanning mode with a Leica TCS-SP5 confocal microscope equipped with an HCX PL APO 100×/1.44NA oil objective. All images were exported from LAS AF (Leica) acquisition software as TIF files. Contrast and brightness were adjusted for illustrative purposes with Adobe Photoshop CS5.

### Animals and Housing Conditions

All behavioral experiments were approved by the local animal care and use committee in accordance with the German animal protection law. For behavioral testing, mice were housed in groups of four to six in standard plastic cages, with food and water ad libitum. Animals were kept under a 12 h light-dark cycle (lights on at 7:00 am) and an ambient temperature of 20–22 °C.

### Behavioral Characterization of Mice

All behavioral experiments were conducted by investigators unaware of the genotype (“blinded”), during the light phase of the day (between 8:00 am and 5:00 pm). *Munc13*-*3* null mutant (−/−) mice are referred to as *Munc13*-*3* ^−/−^ and wild-type (+/+) mice as *Munc13*-*3* ^+/+^. Basic behavioral functions were assessed in two large cohorts of male and female mice which were born around the same time (date of birth with a range of ±4 days). Genders were tested separately (starting with the male cohort, thereafter the female cohort with a delay of 4 weeks) using *Munc13*-*3* ^+/+^ littermate controls. Tests were performed in the following order (see also Table 1 for overview): basic behavior including elevated plus maze (EPM), open field, rotarod, marble burying, visual cliff, grip strength, beam balance, prepulse inhibition (PPI) of the startle response, detailed acoustic startle assessment, and olfaction (buried food test). These basic behavioral tests were conducted first in both male and female mice to ensure that there are no confounding factors that could potentially mislead the interpretation of cognition and social behavior tests. Hence, after this basic behavior characterization, experiments testing higher cognition were performed, which included Morris water maze, hole board working and reference memory task, fear conditioning (in male mice) [23–26], and IntelliCage-based place and multiple reversal learning (in female mice). After cognition tests, social behavior (in male mice) was assessed using a modified version of the social interaction in the tripartite chamber test [27], as well as tests of social interaction in pairs, and nest building. The age of mice at the beginning of testing was 11–12 weeks for male mice and around 16 weeks for female mice. Inter-test interval varied depending on the degree of “test invasiveness” but was at least 24 h.

**Table 1:** Behavioral characterization of male and female *Munc13*-*3* ^−/−^ and *Munc13*-*3* ^+/+^ mice

Males	Start on day	Females	Start on day
Elevated plus maze	1	Elevated plus maze	1
Open field	2	Open field	2
Rotarod	3	Rotarod	4
Marble burying	9	Marble burying	6
Visual cliff	11	Visual cliff	9
Grip strength	12	Grip strength	10
Beam balance	15	Beam balance	11
Prepulse inhibition	18	Prepulse inhibition	17
Startle assessment	19	Startle assessment	18
Buried food finding	22	Buried food finding	34
Hole board	79	IntelliCage	107
Morris water maze	148		
Interaction in pairs	215		
Fear conditioning	219		
Nest building	383		
Tripartite chamber	386		

The experimental day of the testing period on which a specific test was initiated is indicated under the columns with the heading “Start on day.” Males were started at the age of 10–12 weeks and females at 16 weeks

#### Elevated Plus Maze

The apparatus was made of gray Perspex with a central platform (5 × 5 cm), two open and two walled arms (30 × 5 × 15 cm each). Illumination density at the central platform was 135 lux. The mouse was placed on the central platform and was allowed to explore the apparatus for 5 min. The time spent [s] and distance travelled [mm] in the walled and open arms as well as the mean running velocity [s/mm] were measured using an automated tracking software (Viewer2, Biobserve, Germany).

#### Open Field

The mouse was placed into the center of a gray circular Perspex arena (diameter 120 cm; height of wall 25 cm). During a test duration of 7 min, the time spent [s] in the peripheral, intermediate, and center zone of the apparatus and the total distance travelled [mm] as well as the mean running velocity [s/mm] were measured using an automated tracking software (Viewer2, Biobserve, Germany).

#### Rotarod

Rotarod is a test for motor function, balance, and coordination and comprises a rotating drum (ENV-577M, Med Associates Inc. Georgia, Vermont, USA) that is accelerated from 4 to 40 revolutions per minute (rpm) over the course of 5 min. Each mouse was placed individually on a drum and the latency until the mouse slid off the drum was recorded using a trip switch. To assess motor learning, the rotarod test was repeated 24 h later.

#### Marble Burying Test

The mouse was introduced into a cage (34.5 × 56.5 × 18 cm) that contained standard bedding (5 cm fill height). Twenty-four glass marbles were placed on top of the bedding, arranged in six rows with four marbles per row at a distance of 4 cm on top of the bedding. Trial duration was 30 min. The number of marbles buried was registered.

#### Visual Cliff Test

A Perspex box (70 × 35 × 30 cm) that had a transparent floor was placed on the edge of a laboratory bench so that 50 % of its base was positioned on the bench (“ground” side), while 50 % of the base protruded over the edge of the bench, suspended 1 m above the floor (“air” side). The mouse was placed in the middle of the ground side, and the time spent on both sides of the box over a period of 5 min was measured using a video tracking system (Viewer2, Biobserve, Germany).

#### Grip Strength

A grip strength meter (TSE Systems, Bad Homburg, Germany) was used to assess forelimb grip strength. Mice were lifted and held by their tail so that their forepaws could grasp a wire grid. The mice were then gently pulled backward by the tail with their posture parallel to the surface of the table until they released the grid. The peak force applied by the forelimbs of the mouse was recorded in pond. Each mouse performed the test three times, and the average of the three trials was used for statistical analysis.

#### Beam Balance

Beam balance is a sensitive test for (fine) motor coordination (balance) and vestibulomotor function. On the first day, two habituation trials were conducted where mice were placed on an elevated horizontal beam (25 mm in diameter, 59 cm in length), illuminated at the start side and with a dark little cage with bedding at the other end. With the goal to enter the little cage, mice were first placed directly in front of the cage (phase 1) and then in the middle of the beam (phase 2). On the second day, all mice were first habituated again on the 25 mm beam, this time being positioned on the illuminated start. This forces them to cross the whole beam for entering the little cage. For the following test, mice were placed on the start side of a 10 mm beam, and the time needed to pass the beam was recorded. On the third day, mice were again first put on the 25 mm beam and then switched onto an 8 mm beam. If a mouse fell down, the test was repeated (maximally three trials per mouse). If all trials failed, a cutoff time of 60 s was used for calculations. Wild-type animals are able to traverse the 25 mm beam on day 3 within 4.15 ± 0.57 s (male) and 6.74 ± 0.69 s (female) and the 8 mm beam within 10.65 ± 1.3 s (male) and 11.0 ± 1.1 s (female).

#### PPI of the Acoustic Startle Reflex

Mice were placed in small metal cages (82 × 40 × 40 mm) to restrict major movements and exploratory behavior. The cages were equipped with a movable platform floor attached to a sensor that recorded vertical movements of the floor. The cages were placed in four sound attenuating cabinets (TSE Systems, Bad Homburg, Germany). Startle reflexes were evoked by acoustic stimuli delivered by a loudspeaker that was suspended above the cage and connected to an acoustic generator. The startle reaction to an acoustic stimulus that induces a movement of a force-sensitive platform was recorded over a period of 260 ms beginning with the onset of the pulse. An experimental session consisted of a 2 min habituation to a 65 dB background white noise (continuous throughout the session), followed by a baseline recording for 1 min at background noise. After baseline recording, six pulse-alone trials using startle stimuli of 120 dB intensity and 40 ms duration were applied to decrease the influence of within-session habituation. These data were not included in the 120 dB/40 ms analysis of the PPI. For tests of PPI, the startle pulse was applied either alone or preceded by a prepulse stimulus of 70-, 75-, or 80 dB intensity and 20 ms duration. A delay of 100 ms with background noise was interposed between the presentation of the prepulse and pulse stimulus. The trials were presented in a pseudorandom order with a variable interval ranging from 8 to 22 s. The amplitude of the startle response (expressed in arbitrary units) was defined as the difference between the maximum force detected during the recording window and the force measured immediately before the stimulus onset. For each animal, the amplitudes were averaged separately for the two types of trials (i.e., stimulus alone or stimulus preceded by a prepulse). PPI was calculated as the percentage of the startle response using the following formula: %PPI = 100 − [(startle amplitude after prepulse) / (startle amplitude after pulse only) × 100].

##### Startle Response to 120 dB

The response of the mouse to the startle stimulus of 120 dB alone, without any prepulse, as well as the response without stimulus presented was also recorded.

#### Detailed Startle Assessment

In order to assess the integrity of the sensorimotor reflex system that mediates the startle reflex to a sudden loud noise, we determined a detailed *tone intensity*-*startle response curve*. The startle reaction to an acoustic stimulus (pulse) is a short-latency reflex that is mediated by an oligosynaptic neural circuit that includes the lower brainstem, spinal and cranial motor neurons, and the cerebellum [28, 29]. The startle response was measured in a startle box. Here, the startle reflex induces a movement of a force-sensitive platform, which was recorded over a period of 100 ms, beginning with the onset of the pulse. An experimental session consisted of a 2 min habituation period to a 65 dB background white noise, followed by a baseline recording for 1 min. After baseline recording, stimuli of different intensity and a fixed duration of 40 ms were presented. Stimulus intensity was varied between 65 and 120 dB, such that 19 intensities (in steps of 3 dB) from this range were used. Each stimulus intensity was presented ten times in a pseudorandom order with an inter-stimulus interval of 8–22 s. The amplitude of the startle response was determined as described in the “PPI of the acoustic startle reflex” section, with one exception: For each mouse, the amplitudes of the startle responses were averaged for specific stimulus intensities.

#### Buried Food Test

The mouse was habituated to the test cage (29.5 × 18.5 × 13 cm) for three consecutive days with two daily trials of 20-min duration each. On days 4 to 6, the mouse had been food deprived for approximately 22 h prior to the two daily habituation trials and received a piece of chocolate cookie (1.6 g) during each habituation trial and three to five cookies in its home cage after testing. After the second habituation trial, the mouse had access to food in its home cage for 1 h. On day 7, the mouse had to locate a piece of chocolate cookie that was hidden approximately 1.5 cm below fresh bedding close to the wall at one end of the cage. The mouse was placed into the right corner at the opposite end of the cage, and the time [s] it needed to locate the cookie and to start burying to recover it was measured with a cutoff time of 3 min. The mouse was removed from the test cage after the cookie had been discovered and before it was consumed. As a control for possible motivational deficits, a visible test trial was performed after the hidden food test. Again, the latency to locate the cookie was measured with a cutoff of 3 min.

#### Hole Board Paradigm for Working and Reference Memory

The method has been described in detail elsewhere and was applied here with small modifications [30]. Briefly, the test was performed in the hole board chamber used for the assessment of exploratory behavior [23, 31]. Following a habituation period of 6 days, mice were subjected to the acquisition (testing) phase for eight consecutive days, in which their ability to remember which 4 of 16 equidistant holes were baited with sucrose (6 % sucrose, 6 μl) was measured. Each daily session comprised two trials of maximally 5 min duration. The trial was terminated after all rewards were collected or if 5 min time had elapsed, whichever came first. The average number of errors per trial per day was used as measure of cognitive performance, with respect to working and reference memory. A working memory error was defined as re-entering a hole already visited during a specific trial whereas the reference memory error was defined as entering a hole, which was never baited during that testing phase. Furthermore, a learning index for each individual mouse was calculated as follows: total correct response / [(incorrect response + 1) × trial duration in seconds]. After a retention interval of 4 weeks, the mice were tested for long-term memory performance applying the original configuration of baited holes used during the acquisition/testing stage [32]. On the following day, the mice were subjected to a re-acquisition test over four consecutive days. The animals were tested with the same configuration of baited holes as during the initial acquisition and long-term retention test with two trials of 5 min duration. After completion of the re-acquisition test, the configuration of the baited holes was changed. Over four consecutive days (again with two trials of 5 min duration per day), the mice were required to learn a novel configuration of baited holes (reversal learning) which tested their cognitive flexibility and ability to adapt their foraging behavior to a sudden change in the reinforcement contingencies in the testing environment.

#### Morris Water Maze

Spatial learning and memory was additionally assessed in a water maze [33], where spatial learning is based on negative reinforcement as compared to positive reinforcement provided in the hole board working and reference memory test.

A large circular tank (diameter 1.2 m and depth 0.68 m) was filled with opaque water to a depth of 0.6 m. An escape platform (10 × 10 cm) was submerged 1 cm below the surface. The swim trajectory of the mouse was monitored by a computer and the video tracking system Viewer2 (Biobserve, Germany). The escape latency, swim speed, and path length were recorded for each mouse. During the first 2 days, mice were trained to swim to a visible platform (visible platform task) that was marked with a 15 cm-high black flag and placed pseudo-randomly in different locations across trials (non-spatial training). The extra-maze cues were hidden during these trials. After 2 days of visible platform training, hidden platform training (spatial training) was performed. For 8 days, mice were trained to find a hidden platform (i.e., the flag was removed) that was located at the center of one of the four quadrants of the pool. The location of the platform was fixed throughout testing. Mice had to navigate using extra-maze cues that were placed on the walls of the testing room. Every day, mice went through four trials with an inter-trial interval of 5 min. The mice were placed into the pool facing the side wall randomly at one of four start locations and allowed to swim until they found the platform or for a maximum of 90 s. Any mouse that failed to find the platform within 90 s was guided to the platform. The animal then remained on the platform for 20 s before being removed from the pool. The next day after completion of the hidden platform training, a spatial probe trial was conducted. The platform was removed from the pool, and the mice were allowed to swim freely for 90 s.

In order to know whether Munc13-3^−/−^ mice would indeed use an allocentric rather than egocentric search and navigational strategy, we analyzed the spatial probe test regarding the following readouts:

1. The preference for the former platform zone expressed as percent of time spent in the target zone. Mice with intact spatial learning abilities are expected to spend significantly more time in the target zone that formerly contained the hidden platform as compared to the remaining three zones.
2. The latency of the first visit to the target zone and the remaining three zones.
3. The latency of the first visit (crossing) of the former platform location within the target quadrant. The readouts (2) and (3) measure the purposefulness or directionality of the search (in the absence of significant differences in swimming speed). An animal that swims a straight path between entry point and the former platform position would need less time to enter the target zone; it would cross the former platform location with a relatively short latency and would therefore use an efficient allocentric, instead of an egocentric, spatial navigation strategy.
4. The total number of visits to the target zone and the remaining three zones.
5. The total number of visits (crossings) of the former platform location within the target quadrant. An animal that shows similar number of visits to all four zones is likely to use a non-spatial circling strategy that begins at the outer annulus where the maze wall is located and ends at the inner annulus where the former platform location is positioned.
6. The mean swim speed in the target zone and the remaining three zones. An animal that has acquired a spatial memory and is able to navigate through the maze by using extra-maze cues should slow down its swimming velocity specifically in the target zone (if it is indeed searching for the former platform position). Spatial search strategies should be associated with lower swimming velocities in the target zone as compared to the other zones.
7. The total path length (distance swum) in the target zone and the remaining three zones. Animals that have acquired a spatial memory of the former platform position should exhibit a significantly higher distance swum in the target zone as compared to the remaining three zones.

To investigate the flexibility of cognitive processes in mice, the reversal water maze test was performed. The experimental procedure was identical to the one used for the hidden platform training with the exception that the escape platform was moved from the original position to the neighboring quadrant.

#### Cued and Contextual Fear Conditioning

Training consisted of exposing mice for 120 s to the context to assess the baseline level of activity. This period was followed by a 5 kHz 80 dB tone (conditioned stimulus (CS)) for 10 s. Immediately after the tone, a 2 s 0.4 mA foot shock (unconditioned stimulus (US)) was applied. The tone and foot shock (CS-US pairing) were repeated once more after a 10 s resting interval. The second foot shock was followed by 30 s period without any sound/shock; this was done to avoid the formation of an aversive association to the handling procedure in the mouse. Mice were trained within the same session for both contextual and cued fear conditioning. The contextual memory test was performed 24 h after the CS-US training. Mice were monitored for 120 s for freezing in the same context as used for training without any tone or foot shock. The cued memory test was performed 4 h after the contextual memory was tested in a new chamber. First, mice were monitored for freezing over a 120 s precue period with no tone to assess freezing in the new context. Next, a 120 s cue period followed in which the tone was presented. Duration of freezing behavior, defined as absolute lack of movement (excluding respiratory movements), was recorded by a video camera and a PC equipped with “Video Freeze” software (MED Associates, St. Albans, Vermont, USA).

#### Nest Building

The mouse was single-housed 1 h before lights were turned off. The cage contained bedding material and nesting towels. After two nights of habituation, nesting towels were replaced by nestlets (pressed cotton squares weighing ∼3 g). Nest building was assessed on the next morning. The remainder/leftover of the nesting material was weighed, and the proficiency of nest building was rated using a scale that ranged from 1 to 5 with lower scores indicating poor nest building behavior.

#### Social Interaction in Pairs

Every mouse was first individually habituated to the testing cage (30 × 30 × 30 cm) for 10 min over two consecutive days. On day 3, pairs of unfamiliar mice of the same genotype were placed into the testing cage for 10 min. The time that the animals spent in close contact [s] was recorded by a trained observer who was unaware of the genotype of the mice.

#### Sociability and Social Memory

The tripartite chamber [27] was a rectangular box that was divided into three chambers (40 × 20 × 22 cm). The dividers were made from transparent Plexiglas and had rectangular entries (35 × 220 mm). The floor of the box was covered with bedding that was exchanged between trials. The test mouse was introduced into the middle chamber, with the entries to the other two chambers closed, and allowed to acclimatize for 5 min. Thereafter, a small wire cage (140 × 75 × 60 mm) containing an unfamiliar male C57BL/6N mouse of the same age and weight (stranger 1) was placed in one outer chamber. An empty wire cage was positioned in the other outer chamber. The location (outer left or right chamber) of stranger 1 was alternated between trials. After unblocking the entries to the outer chambers, the test mouse was allowed to freely move between chambers for 10 min. The time spent in and number of entries into each chamber were recorded by a video tracking system (Viewer2, Biobserve GmbH, Germany). Each mouse received second and third trials. The second trial was identical to the first trial except that the stranger mouse was placed into the other outer chamber in order to control for a possible side bias. On the third trial, the test mouse was presented with the familiar stranger 1 and an unfamiliar stranger 2. Sociability and social memory indexes were calculated as follows: Sociability index = [time investigating stranger / time investigating stranger + time investigating empty cage] × 100; Memory index = [time investigating unfamiliar mouse / time investigating unfamiliar + familiar mouse] × 100.

#### IntelliCage

The fully automated high-throughput IntelliCage^R^ system (in the following called IntelliCage) has been described previously [34, 35]. The IntelliCage paradigm allows to measure mouse cognitive behavior with a minimum of experimenter intervention and in an ecological social environment (up to 16 mice can be tested simultaneously in one IntelliCage), i.e., in the presence of other animals (as compared to other behavioral paradigms where animals are tested individually). Described in detail here is the protocol developed specifically for this study. A maximum of 14 mice, with both genotypes present (random assignment), were tested at once in the IntelliCage. The experimental design consisted of four different stages that are explained in more detail under the “Experimental Stages” section. In preparation of the IntelliCage test, the animals were subcutaneously injected with a transponder for telemetric individual identification. At 24 h later, mice were placed into the IntelliCages for a habituation session of 24 h. Thereafter, mice received nose poke training for five consecutive days. After completion of the nose poke training, they were tested over seven consecutive days in a place learning task. Subsequent to the place learning task, mice were tested in a multiple reversal task over nine consecutive days.

##### Transponders

At the age of 8 weeks, mice were anesthetized with an intraperitoneal injection of 0.25 % tribromoethanol (Sigma, Steinheim, Germany) at a dose of 0.125 mg/g. Under anesthesia (5-min postinjection), they received a subcutaneous implantation of an ISO standard transponder (PM162-8) with a length of 8.5 mm and a diameter of 1.2 mm below the skin of the neck, using a specific injection device. One day after transponder injection, mice were placed into the IntelliCage.

##### Apparatus

The IntelliCage (TSE Systems, Bad Homburg, Germany) apparatus is placed inside a standard laboratory rodent cage (height 20.5 cm, length 55 cm, width 38.5 cm; Techniplast Model 2000). The cage floor is covered with sawdust bedding and contains four housing shelters just beneath the food hopper. The apparatus consists of four right-angle triangular conditioning chambers (15 × 15 × 21 cm) that are located in the corners of the cage. The mouse has access into the conditioning chambers via a plastic tube (30-mm diameter) which contains a ring RFID antenna that identifies the specific animal via the detection and readout of the implanted transponder. Each corner contains a differential temperature detector that detects the presence of a mouse. Additionally, each conditioning chamber contains two motor-driven doors that can be opened by the mouse if it disrupts one of two light barriers with a nose poke. Each door then gives access to one of two water bottles present in each corner. The cap of each water bottle is equipped with a lickometer that allows measuring the number of licks made during a visit. Each corner also contains two rows of three indicator LEDs. The IntelliCage is connected to a PC software for designing experiments, controlling data acquisition, online monitoring, and data collection.

##### Experimental Stages

One day after the implantation of the transponders, the animals were placed into the IntelliCage where they remained for 22 days until the end of the experiments. Cage bedding was changed once weekly after the conclusion of an experimental stage.

##### Habituation

During the habituation session (24 h), the mice had free access to all four corners with the eight water bottles. During this session, the animals learned that water is available in the corners of the IntelliCage.

##### Nose Poke Training

Nose Poke training was performed over five consecutive days. During this stage, the doors that give access to the water bottles were closed and opened only upon activation of the nose poke sensor. Thus, the animals had to learn that they could open the door to a water bottle with a nose poke.

##### Place Learning

During the 7 days of place learning, the mice learned that only one corner provides access to water bottles, while the other three corners were blocked and the doors could not be opened by nose pokes. The specific corner where a specific mouse could drink was determined by the least preferred corner during the nose poke training stage. The baseline corner preference during the nose poke training was determined as the percentage of visits to one specific corner divided by the total number of visits to all four corners and multiplied with 100. For example, if a mouse showed the following preference pattern during the nose poke training stage: corner1 35 %, corner2 11 %, corner3 26 %, and corner4 28 % visits, then the rewarded corner chosen for the place learning stage would be corner2.

##### Multiple Reversals

After completion of the place learning stage, the mice were subjected to a multiple reversal learning test that allows to measure cognitive flexibility or perseveration, respectively. Over nine consecutive days, the mice received three tests of reversal learning, each lasting 3 days. On the first reversal test, the mice had to learn that the corner that was rewarded during the place learning stage was blocked, while water was now available in the corner that was least preferred during the place learning stage. On the reversal tests 2 and 3, the animals had to re-learn again the current location of the accessible water bottles, with the corner being rewarded that was least preferred during the previous reversal test.

### Statistical Analysis

All data were analyzed separately for males and females. Between-group comparisons were made by either one-way analysis of variance (ANOVA) with repeated measures or *t* test for independent samples. Within-group comparisons were made via *t* tests for dependent samples. Within-group tests of chance level performance using ratio or percentage calculations were performed via single group *t* tests against a chance level of either 0.25 or 0.5 when indicated. Mann-Whitney *U* and Wilcoxon tests were used if the normality assumption was violated (as assessed by the Kolmogorov-Smirnov test). All statistics were performed using SPSS v.17 (San Diego, USA) or Prism GraphPad software. Data presented in the figures and text are expressed as mean ± SEM; *p* values <0.05 were considered significant.

## Results

### Munc13-3 is Expressed in Both Cerebellum and Hippocampal Dentate Gyrus in 8- and 14-Week-Old Mice

**Fig. 1:**
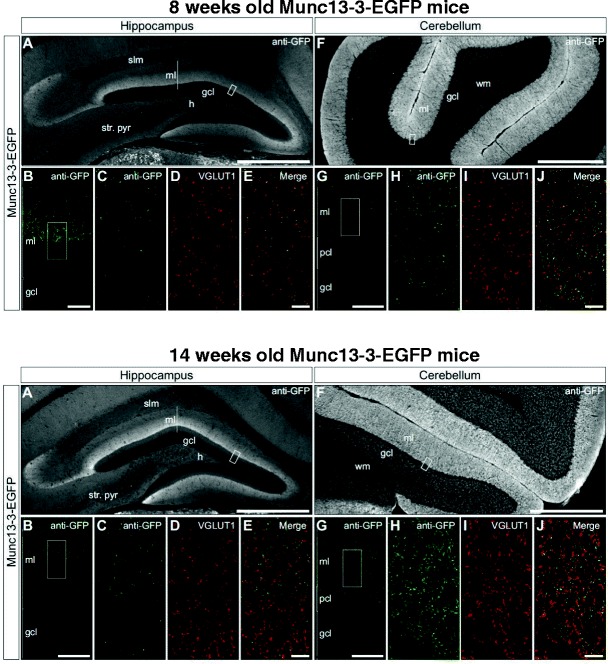
Immunolocalization of Munc13-3 in hippocampus and cerebellum. *Upper row*—8-week-old Munc13-3-EGFP mice. *Lower row*—14-week-old Munc13-3-EGFP mice. Munc13-3 immunoreactivity in the hippocampus (**A**–**E**) and cerebellum (**F**–**J**) is revealed by immunodetection of GFP in 8- and 14-week-old Munc13-3-EGFP mice. The expression pattern of the Munc13-3-EGFP signal is similar in both age groups. In the hippocampus, the pattern of GFP immunoreactivity is consistent with the expression of Munc13-3 in perforant path inputs to the molecular layer of the dentate gyrus and the stratum lacunosum-moleculare of the CA3 subregion. **B**, **G** Single-plane confocal micrographs of regions depicted by *white boxes* (**A**, **F**) illustrate that Munc13-3-EGFP signal is restricted primarily to the central and outer laminae of the dentate gyrus molecular layer in the hippocampus (**B**) and to granule cell and molecular layers in the cerebellum (**G**). **C**–**E**, **H**–**J** In the dentate gyrus (**C**–**E**) and the cerebellum (**H**–**J**), dual labeling confocal microscopy reveals frequent colocalization of Munc13-3-EGFP (**C**, **H**) and VGLUT1 (**D**, **I**) signals, as seen in the merged panels (**E**, **J**), indicating that Munc13-3 is primarily localized to glutamatergic presynaptic terminals. *gcl* granule cell layer, *pcl* Purkinje cell layer, *ml* molecular layer, *h* hilus, *slm* stratum lacunosum-moleculare, *str. pyr* stratum pyramidale, *wm* white matter. *Scale bars*: **A**, **F**, 500 μm; **B**, **G**, 20 μm; **E**, **J**, 5 μm

Immunohistochemical detection of Munc13-3-EGFP revealed specific labeling in both the hippocampal dentate gyrus (Fig. 1A–E) and the cerebellum (Fig. 1F–J) of 8-week-old (upper row in Fig. 1) and 14-week-old (lower row in Fig. 1) mice. The expression pattern and the intensity of the Munc13-3-EGFP signal were similar between 8- and 14-week-old Munc13-3-EGFP mice. The specificity of this approach was validated by the absence of immunofluorescent signals in identically treated sections from wild-type animals (data not shown). Whereas the expression of Munc13-3 in the cerebellum has been previously described [16, 36], we provide here the first evidence that Munc13-3 protein is also targeted to a subset of presynaptic terminals in the hippocampus. Munc13-3-EGFP immunoreactivity in the hippocampus was restricted to the middle and outer laminae of the dentate gyrus molecular layer, consistent with its presence in perforant path inputs projecting from the entorhinal cortex to the distal dendrites of granule cells [37], but was conspicuously absent from commissural and associational inputs to the inner-most lamina of the molecular layer. Dual labeling confocal microscopic analyses demonstrated that Munc13-3-EGFP and VGLUT1 signals frequently colocalize in both the dentate gyrus (upper and lower rows in Fig. 1C–E) and the cerebellum (upper and lower rows in Fig. 1H–J), indicating that Munc13-3 is subcellularly targeted to presynaptic terminals in a subset of glutamatergic neurons. Of note, Munc13-3-EGFP signals in perforant path inputs to the dentate gyrus were significantly weaker than Munc13-3 detected in the cerebellum, perhaps accounting for the absence of a Western blot signal in hippocampal homogenates probed with Munc13-3-specific antibodies [16].

### Sensory Functions, Activity, and Anxiety Are Unaffected in *Munc13*-*3* Null Mutants

**Fig. 2:**
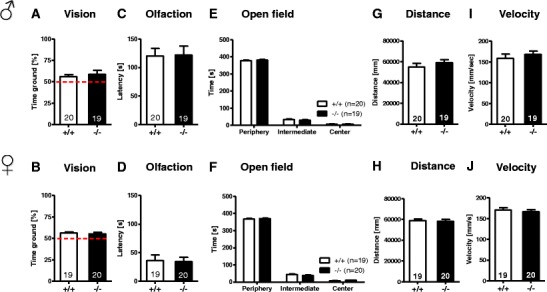
Basic behavioral characterization of *Munc13*-*3* ^−/−^ mice versus wild-type littermates of both genders reveals no differences between the genotypes. The *upper panel* represents male and the *lower panel* female mice. **A**, **B** Vision: visual cliff test. **C**, **D** Olfaction: buried food test. **E**–**J** Activity: open field readouts. **E**, **F** Time spent in various zones. **G**, **H** Total distance travelled. **I**, **J** Average velocity. Mean ± SEM presented; respective sample sizes are indicated in the panels

Sensory functions, i.e., vision (visual cliff test) and olfaction (buried food test), were comparable between *Munc13*-*3* ^−/−^ and *Munc13*-*3* ^+/+^ mice, with females being faster in food finding (Fig. 2A–D). A similar difference between male and female in the absence of a genotype difference had been reported for *Gpm6B*-deficient mice and their wild-type littermates [38]. There is also evidence that female mice outperform male mice in odorant detection tasks [39]. This finding has been related to the lower expression of odorant-binding protein genes in the olfactory epithelium of male as compared to female mice [39].

**Fig. 3:**
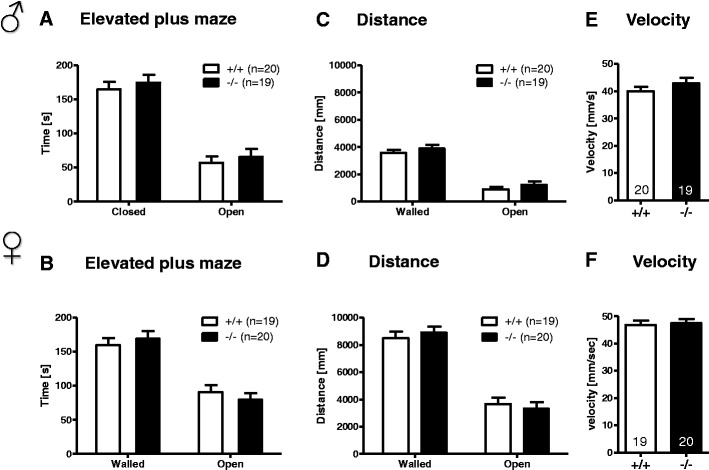
Anxiety-like behavior is not affected by *Munc13*-*3* deficiency. The *upper panel* represents male and the *lower panel* female mice. **A**–**F** Anxiety-like behavior in the elevated plus maze. **A**, **B** Sojourn times in the walled and open arms. **C**, **D** Distance travelled on walled and open arms. **E**, **F** Running velocity. Mean ± SEM presented; respective sample sizes are indicated in the panels

General activity, tested in the open field, yielded similar results for time spent in various zones, distance traversed, and average velocity in both genotypes and genders (Fig. 2E–J). Also, anxiety levels, as evaluated by EPM, did not differ between genotypes in terms of time spent in walled versus open arms, the running speed, or the distance travelled on walled or open arms (Fig. 3A–F). In sum, basic behavioral characterization of both male and female mice revealed no differences between *Munc13*-*3* ^−/−^ and their *Munc13*-*3* ^+/+^ counterparts.

### *Munc13*-*3*^−/−^ Mice Show a Normal Motor Phenotype But Reduced Acoustic Startle

**Fig. 4:**
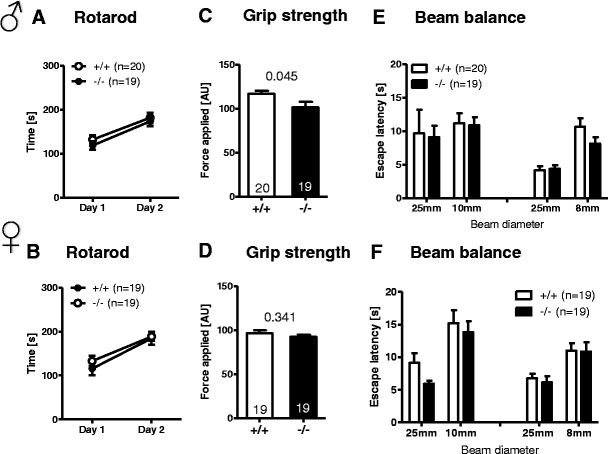
*Munc13*-*3* ^−/−^ mice display a generally normal motor phenotype, except of a marginal reduction in grip strength in male mice only. The *upper panel* represents male and the *lower panel* female mice. **A**, **B** Rotarod. **C**, **D** Grip strength. **E**, **F** Beam balance. Mean ± SEM presented; respective sample sizes are indicated in the panels. *p* values refer to *t* tests for independent samples

Detailed characterization of motor performance in *Munc13*-*3* ^−/−^ mice did not reveal any gross motor phenotype. Motor coordination and learning as assessed by the short routine 2-day rotarod test were comparable between male and female *Munc13*-*3* ^−/−^ and *Munc13*-*3* ^+/+^ mice (Fig. 4A, B). In contrast, a more challenging 22-session rotarod paradigm with very high rotation speeds did detect earlier a motor learning defect in *Munc13*-*3* ^−/−^ mice [17]. Grip strength was slightly reduced in male *Munc13*-*3* ^−/−^ mice as compared to male *Munc13*-*3* ^+/+^ controls (*p* = 0.045, *t* test for independent samples; Fig. 4C). No such difference was observed in the female groups (Fig. 4D).

**Fig. 5:**
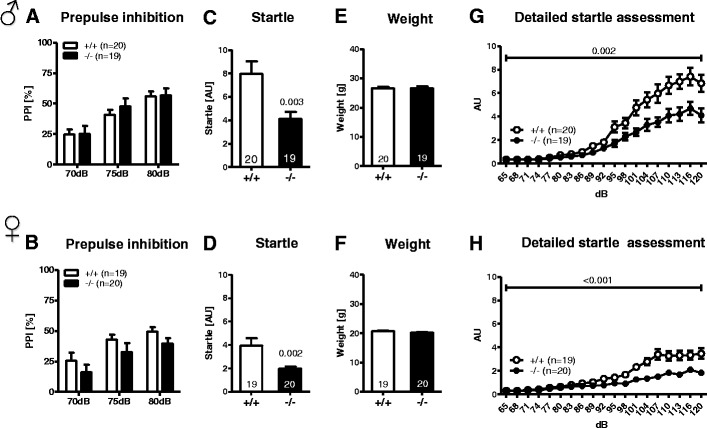
Prepulse inhibition is not affected by *Munc13*-*3* deficiency, but acoustic startle responses are significantly reduced in both male and female *Munc13*-*3* ^−/−^ mice. The *upper panel* represents male and the *lower panel* female mice. **A**, **B** Prepulse inhibition of the acoustic startle reflex (PPI). **C**, **d** Startle response to a 120-dB stimulus. *p* values refer to *t* tests for independent samples. **E**, **F** Body weight at the time of PPI testing. **G**, **H** Detailed assessment of the acoustic startle reflex over a broad range of stimulus intensities. *p* values refer to the main effect of genotype obtained with repeated measures ANOVA. Mean ± SEM presented; respective sample sizes are indicated in the panels

Furthermore, in the beam balance test of motor coordination, no significant differences between genotypes and genders were seen (Fig. 4E–F). Sensorimotor gating, determined by PPI, showed comparable results between genotypes and genders (Fig. 5A, B). Body weight as important control variable in this test did not differ among genotypes (Fig. 5E, F). However, there was a significant reduction in the acoustic startle response to 120 db in *Munc13*-*3* ^−/−^ male (*p* = 0.003, *t* test for independent samples; Fig. 5C) and female mice (*p* = 0.002, *t* test for independent samples; Fig. 5D). Prompted by this finding, we performed a detailed assessment of a tone intensity-startle response curve across a broad range of 65- to 120-dB stimulus intensities. Both male and female *Munc13*-*3* ^−/−^ mice showed a significant reduction in their startle response over a broad range of stimulus intensities, especially at higher stimulus intensities, as compared to their wild-type littermates (males *p* = 0.002; females *p* < 0.001; repeated measures ANOVA; Fig. 5G, H). Apart from the robust decrease of acoustic startle in both male and female *Munc13*-*3* ^−/−^ mice (and a slight reduction in grip strength in male *Munc13*-*3* ^−/−^ mice only), we did not observe any significant motor deficiency.

### *Munc13*-*3*^−/−^ Mice Reveal No Abnormalities in Hippocampus-Related Cognition

#### Morris Water Maze

**Fig. 6:**
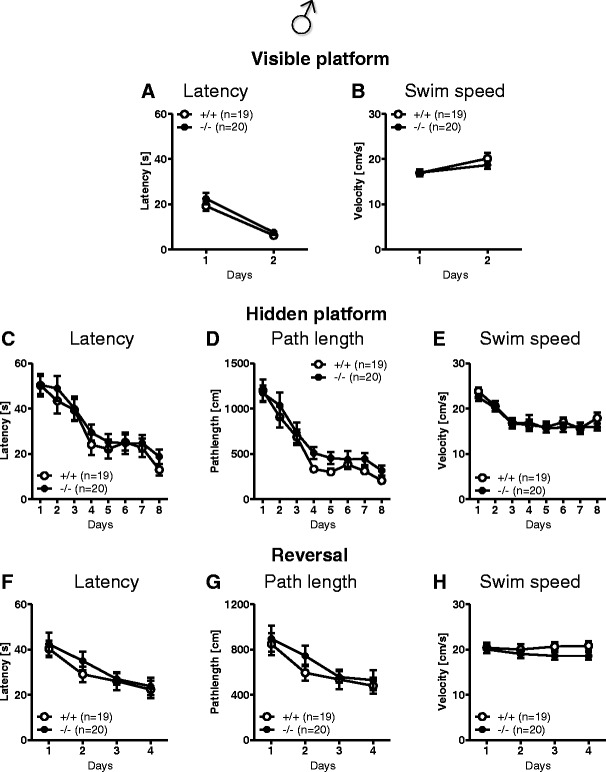
Male *Munc13*-*3* ^−/−^ mice show no cognitive deficits in the Morris water maze. Visible platform test: **A** escape latency and **B** swim speed. Hidden platform test: **C** escape latency, **D** path length, and **E** swim speed. Reversal learning test: **F** escape latency, **G** path length, and **H** swim speed. Mean ± SEM presented; respective sample sizes are indicated in the panels

**Fig. 7:**
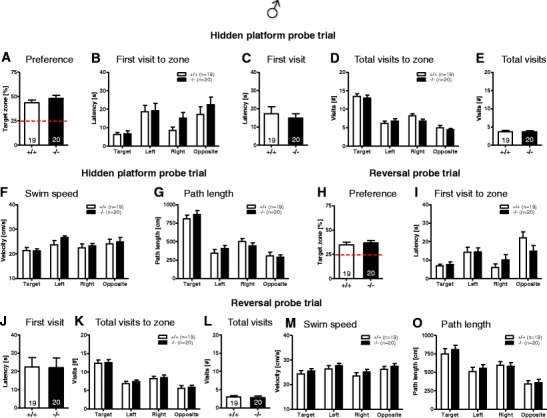
Spatial probe trial behavior is unaffected in male *Munc13*-*3* ^−/−^ mice. Male *Munc13*-*3* ^−/−^ mice use spatial search and navigational strategies similar to *Munc13*-*3* ^+/+^ mice in the hidden platform (**A**–**G**) and reversal learning spatial probe trial (**H**–**O**). **A**, **H** Preference for the former platform zone expressed as percent of time spent in the target zone. *Dashed red line* indicates performance at chance level. **B**, **I** Latency of the first visit to the indicated zones. Please note that (irrespective of genotype) the latency to visit the target zone is significantly lower (*p* values <0.05; *t* test for dependent samples) as compared to the other zones. **C**, **J** Latency of the first visit (crossing) of the former platform location within the target quadrant. **D**, **K** Total number of visits to the indicated zones. Please note that (irrespective of genotype) the number of visits to the target zone is significantly higher as compared to the other zones (*p* values <0.05; *t* test for dependent samples). **E**, **L** Total number of visits (crossings) of the former platform location within the target quadrant. **F**, **M** Mean swim speed in the indicated zones. **G**, **O** Total path length (distance swum) in the indicated zones. Please note that (irrespective of genotype) the path length in the target zone is significantly higher as compared to the other zones (*p* values <0.05; *t* test for dependent samples). Mean ± SEM presented; respective sample sizes are indicated in the panels

In the visible platform as well as in the hidden platform and reversal learning version of the task, mice of both genotypes performed similarly (Fig. 6A–H). Furthermore, no significant differences between genotypes were observed during the spatial probe trials for the hidden platform and reversal learning tests (Fig. 7A–O). A detailed analysis of the search pattern and navigational behavior during the spatial probe trials indicated that *Munc13*-*3* ^−/−^ mice, similar to *Munc13*-*3* ^+/+^ controls, used an allocentric rather than egocentric search strategy to navigate to the target zone and the former platform location. In conclusion, *Munc13*-*3* ^−/−^ mice are able to solve spatial problems in the Morris water maze test without using a non-spatial search and navigation strategy.

#### Hole Board Working and Reference Memory Task

**Fig. 8:**
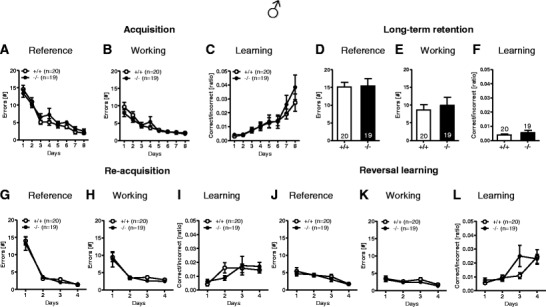
Male *Munc13*-*3* ^−/−^ mice show no learning and memory deficits in the hole board working and reference memory test. Acquisition/training stage: **A** reference memory errors, **B** working memory errors, and **C** learning index. Long-term retention test after a delay of 4 weeks: **D** reference memory errors, **E** working memory errors, and **F** learning index. Re-acquisition test: **G** reference memory errors, **H** working memory errors, and **I** learning index. Reversal learning/cognitive flexibility: **J** reference memory errors, **K** working memory errors, and **L** learning index. Mean ± SEM presented; respective sample sizes are indicated in the panels

This task tests spatial reference memory as well as working memory. The number of working and of reference memory errors in learning phase, re-acquisition phase, and re-learning phase as well as the learning index showed no significant differences between genotypes (Fig. 8A–L).

#### Fear Conditioning

**Fig. 9:**
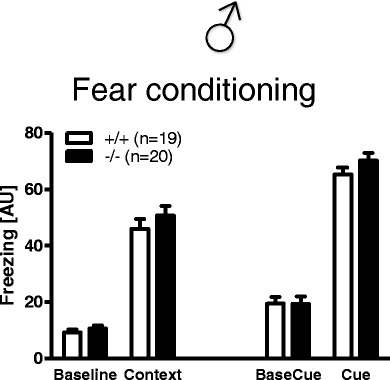
Fear conditioning is unaffected in male *Munc13*-*3* ^−/−^ mice. Context and cued fear conditioning in male *Munc13*-*3* ^−/−^ and *Munc13*-*3* ^+/+^ mice. Mean ± SEM presented; respective sample sizes are indicated in the panels

Contextual fear memory depends on the hippocampus and cued memory also on the amygdala. In all test conditions, baseline, context, precue, and cue, *Munc13*-*3* ^−/−^ mice froze to the same extent as *Munc13*-*3* ^+/+^ mice (Fig. 9). The ability to form fear memories is therefore not disrupted in Munc13-3 deficiency.

#### IntelliCage—Place Learning

**Fig. 10:**
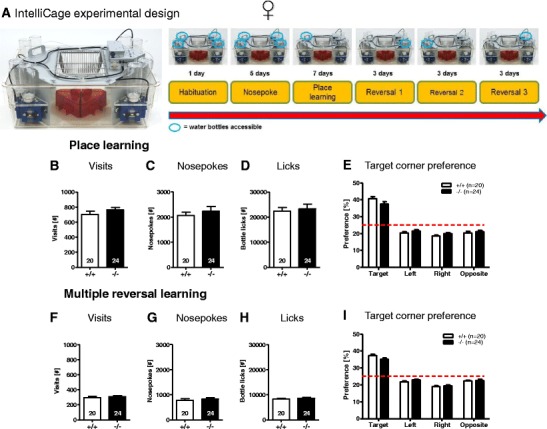
IntelliCage-based cognitive performance is entirely normal in female *Munc13*-*3* ^−/−^ mice. IntelliCage testing in female *Munc13*-*3* ^−/−^ and *Munc13*-*3* ^+/+^ mice. **A** Experimental design: IntelliCage with four conditioning corners (*blue*), four water bottles, four housing shelters (*red*), and food hopper (*left*). The cartoon illustrates the succession of the different experimental stages of the IntelliCage cognitive test battery. The *blue circles* indicate an example of a possible succession of conditioning corners, where a specific mouse could obtain water dependent on its performance in the previous stage of the test. The number of training days for each of the experimental phases is indicated (*right*). General activity and water intake measures across the 7 days of place learning: **B** corner visits, **C** nose pokes, and **D** bottle licks. **E** Target corner preference during the acquisition stage. General activity and water intake measures across the 9 days of multiple reversal learning: **F** corner visits, **G** nose pokes, and **H** bottle licks. **I** Corner preference during the multiple reversal stage. Mean ± SEM presented; respective sample sizes are indicated in the panels

The experimental design is displayed in Fig. 10A. General activity in terms of total corner visits, number of nose pokes, and the number of bottle licks was not significantly different between the *Munc13*-*3* ^−/−^ and *Munc13*-*3* ^+/+^ mice (Fig. 10B–D). These results suggest that both groups showed similar levels of activity during the days of place learning and were equally motivated to drink, suggesting that Munc13-3 deficiency has no significant effect on activity in a home cage setting and water intake. Across the 7 days of place learning, both *Munc13*-*3* ^+/+^ and *Munc13*-*3* ^−/−^ mice developed a significant place preference for the target corner as compared to the remaining three corners (target versus left, right or opposite, all *p* < 0.001; *t* test for dependent samples; Fig. 10E). However, there was no significant difference between genotypes in preference for the four corners or the number of visits to specific corners (data not shown). We also investigated the speed of the acquisition of the place preference during the first night of acquisition by analyzing the number of visits to the target corner as well as the target corner preference in 2 h intervals. Again, no significant difference was observed between *Munc13*-*3* ^−/−^ and *Munc13*-*3* ^+/+^ mice (data not shown). Both genotypes showed a significant above chance level preference for the target corner during the last 2 h of the first night (*Munc13*-*3* ^+/+^ mice 43.7 ± 3.86 %; *p* < 0.001; *Munc13*-*3* ^−/−^ 40.67 ± 2.61 %; *p* < 0.001; single-group *t* test against a chance level of 25 %). These results suggest that place learning in the IntelliCage is not affected by Munc13-3 deficiency.

#### IntelliCage—Multiple Reversals

We next investigated whether *Munc13*-*3* ^−/−^ mice would show changes in cognitive flexibility, respectively perseveration, after three reversal learning challenges. Similar to the findings of the place learning stage, the general activity (total number of visits, nose pokes, and bottle licks) during the 9 days of multiple reversal learning was not significantly different between the *Munc13*-*3* ^−/−^ and *Munc13*-*3* ^+/+^ mice (Fig. 10F–H). Furthermore, both *Munc13*-*3* ^+/+^ and *Munc13*-*3* ^−/−^ mice developed a significant place preference for the target corner as compared to the remaining three corners (target versus left, right or opposite, all *p* < 0.001; *t* test for dependent samples; Fig. 10I). Again, there was no significant difference between genotypes in terms of preference for the four corners or number of visits to specific corners (data not shown). These results suggest that Munc13-3 deficiency has no detrimental effect on cognitive flexibility or the ability to show adaptive behavior to sudden changes in the reinforcement contingencies in the environment.

### *Munc13*-*3*^−/−^ Mice Are Completely Normal in All Relevant Social Behavior Tests

**Fig. 11:**
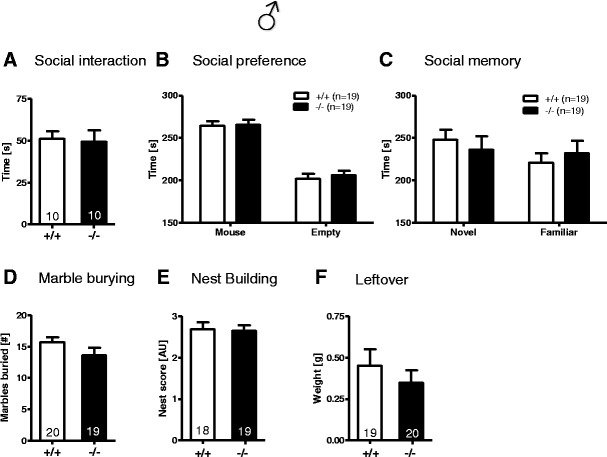
Male *Munc13*-*3* ^−/−^ mice exhibit normal social behavior. These tests were all conducted in male mice. **A** Social interaction in pairs. **B** Social preference. **C** Social memory. **D** Marble burying. **E** Nest building. **F** Leftover of nesting material. Mean ± SEM presented; respective sample sizes are indicated in the panels

One of the tests employed for testing social memory was a modified version of the tripartite chamber [27]. Social interaction in pairs did not reveal genotype differences between *Munc13*-*3* ^−/−^ and *Munc13*-*3* ^+/+^ mice (Fig. 11A). In the tripartite chamber, the *Munc13*-*3* ^−/−^ mice preferred a live mouse over an inanimate empty box to the same extent as *Munc13*-*3* ^+/+^ mice (Fig. 9B). Also, there was no genotype difference in the capability to distinguish between familiar and stranger mouse (social memory; Fig. 11C). Marble burying, measuring compulsive behavior, and nest building, assessing aspects of social competence, were both indistinguishable between genotypes (Fig. 11D–F). Hence, it can be concluded that the *Munc13*-*3* ^−/−^ mice have no deficits in social interaction or skills required for normal social interaction.

## Discussion

Recent studies have shown that the cerebellum shapes the hippocampal spatial code, indicating an important functional connection between cerebellum and hippocampus [11]. In this context, Munc13-3 could, in principle, play an important regulatory role. It is strongly expressed in the cerebellum [17], where it codetermines the transmitter release probability and short-term plasticity features of granule cell synapses targeting Purkinje, Golgi, and basket cells [17–19], and it is also detectable—as reported here for the first time—in distinct areas of the dentate gyrus of the hippocampus. In view of this expression pattern and these functional characteristics, we pursued the idea that Munc13-3-dependent mechanisms could contribute to the functional link between cerebellum and hippocampus. To this end, we conducted a comprehensive behavioral characterization of *Munc13*-*3* mutant mice, combining tests of hippocampus-dependent spatial cognition with basic and cerebellar behavioral readouts.

Apart from a slight reduction in grip strength of male *Munc13*-*3* ^−/−^ mutants, *Munc13*-*3* ^−/−^ and wild-type mice of both genders did not differ in any basic or cognitive tests. The only remarkable observation that we made in *Munc13*-*3* ^−/−^ mice was a robust reduction in the acoustic startle response, a reflex process that has been linked to cerebellar dysfunction [28]. The impaired acoustic startle reflex in *Munc13*-*3* ^−/−^ mice is not paralleled by an altered PPI, which can be explained by the fact that the level of PPI seems to be independent of the magnitude of the startle response, indicating that sensorimotor gating processes in mice are not necessarily linked to startle reactivity [40].

Similar reductions in the acoustic startle response as seen in *Munc13*-*3* ^−/−^ mice were previously observed in numerous mouse models with very different genetic lesions and consequent functional defects, including, for example, mice lacking the 5-HT1B receptor [41], Parkin [42], GLAST [43], TAK1 [29], or phenylethanolamine *N*-methyltransferase [44]. In all these cases, however, the genetic lesions cause multiple additional behavioral deficits, which is not the case with the Munc13-3 deletion and likely due to the more widespread and/or pleiotropic functional deficits caused by the respective mutations. TAK1 deficiency, for instance, is associated with delayed cerebellar growth, a smaller cerebellum, and a disruption of lobules VI–VII [29]. Correspondingly, *TAK* ^−/−^ mice do exhibit not only a reduced acoustic startle response but also deficits in social interaction, which was not observed in *Munc13*-*3* ^−/−^ mice. Similarly, *Tsc1* ^−/−^ mice exhibit age-dependent Purkinje cell loss as well as morphological changes and reduced excitability of Purkinje cells [45], along with an autism spectrum disorder-like social interaction phenotype. In contrast to *TAK* ^−/−^ and *Tsc1* ^−/−^ mice, *Munc13*-*3* ^−/−^ mutants show a normal cerebellar cytoarchitecture [17] and unaltered social behavior, indicating that cerebellar neurodegeneration and morphological changes are more critical for the expression of altered social behaviors than the changes observed in cerebellar neurotransmission and short-term plasticity at granule cell synapses targeting Purkinje, Golgi, or basket cells of *Munc13*-*3* ^−/−^ mutant mice.

At the molecular level, the fact that Munc13-3 loss in the cerebellum has only subtle synaptic and behavioral consequences is probably due to the continued presence of Munc13-1 in the cerebellum, which can likely supplant Munc13-3 function in many respects. The reasons for the rather selective effect of Munc13-3 loss on the startle response (present study) can be manifold. The startle reaction to an acoustic stimulus is a short-latency reflex (5 ms in the neck, 8 ms in the hindleg) [46]. It is mediated by an oligosynaptic neural circuit that includes the lower brainstem, spinal, and cranial motor neurons and the cerebellum and is therefore critically dependent on synchronous, fast, and reliable synaptic transmission to achieve the required short latency. A plausible cause for the rather selective effect of Munc13-3 loss on the startle response might therefore be that the Munc13-3 deletion causes very selective defects in short-term synaptic plasticity within the cerebellum while leaving overall functional network connectivity intact [17–19]. This altered short-term plasticity, in turn, is predicted to cause changes in cortical gain control [47], motor control [48], or sensory adaptation [49] and might thereby affect the acoustic startle response. This notion is supported by the fact that Purkinje cell degeneration (pcd) mice also show a reduced startle response [50], which indicates that synaptic transmission and plasticity at granule cell to Purkinje cell synapses can modulate the acoustic startle response, as seems to be the case with *Munc13*-*3* ^−/−^ mice. In addition, it is possible that the functional synaptic changes caused by Munc13-3 loss are too subtle to be detectable at the behavioral level by most of the currently established behavioral assays. Finally, it is feasible that the cerebellar network can counteract subtle synaptic defects caused by the loss of Munc13-3 independently of other Munc13s, e.g., by fine-tuning synaptic efficacy and short-term plasticity within the network in a homeostatic manner.

Beyond analyses of general and cerebellum-related behavioral readouts, we employed a comprehensive cognitive test battery to gain insight into the effects of *Munc13*-*3* deletion in overall cognition. Toward this end, a series of tests, including Morris water maze, hole board working and reference memory task, fear conditioning, and a newly established IntelliCage-based test for cognitive flexibility, were conducted. The spatial probe trials in the Morris water maze test indicated that *Munc13*-*3* ^−/−^ mice solved the water maze problems mainly through the use of spatial/allocentric search strategies. It remains to be determined whether the *Munc13*-*3* ^−/−^ mice would show impairments in the Morris water maze test if tested under infrared light conditions [11], where the mice are forced to use egocentric search strategies. In essence, the *Munc13*-*3* ^−/−^ mice performed all learning and memory tests with the same proficiency as their WT counterparts. Even fear conditioning data showed no genotype differences in both cued and contextual fear memory, although previous studies, e.g., on hotfoot mice [51], which have a primary deficiency of parallel fiber to Purkinje cell synapses, displayed deficits in cued fear conditioning.

IntelliCage experiments allowed us to study the cognitive flexibility of the mice in a more robust way [34, 35, 52]. For instance, the corner where a specific mouse could drink was determined by the least preferred corner during the nose poke training stage. This kind of customization for each mouse allowed us to monitor the learning proficiency of every mouse individually and gauge its performance in the learning phase and through the multiple reversal phases. Even with a highly sensitive test like this, *Munc13*-*3* ^−/−^ mice learnt the paradigm with the same ease as WT mice. Multiple reversal phases were implemented to challenge the memory load of the mouse [53], forcing it to re-learn new reinforcement contingency for each of the three reversal phases. This allowed us to assess the limit to which the mouse can handle the conflict between an existing memory for a specific association of reward with a specific hole and a new memory, which teaches the mouse to reject the previously learnt association [54]. This forces its memory system to actively form a new memory for the most recently baited hole and suppress the previous memory [54]. The *Munc13*-*3* ^−/−^ mice showed the same level of cognitive flexibility as the WT mice and succeeded with the learning of new reward locations throughout the reversal phases even as the task got more cognitively demanding with each reversal.

It has been proposed that the cerebellum plays an important role in procedural learning (i.e., the learning of the basic task requirements and the general rules of the task) during the initial stages of learning a complex task including spatial learning in the Morris water maze or radial arm maze tasks [8, 10, 55, 56]. However, early stages of Morris water maze, hole board working and reference memory, or IntelliCage testing were very similar between *Munc13*-*3* ^−/−^ and WT mice. These results indicate that even procedural learning is not compromised in *Munc13*-*3* ^−/−^ mice.

Beyond the cerebellum-related behavioral readouts discussed above, the results obtained in the present study show that Munc13-3 deficiency does not cause any aberrations in overall behavior in terms of general activity, cognition, and social functions. As was argued above in the context of the very specific startle response deficit in *Munc13*-*3* ^−/−^ mice, the apparent lack of cognitive defects in the *Munc13*-*3* ^−/−^ mutants may be due to the fact that the continued expression of Munc13-1 and Munc13-2 completely replaces Munc13-3. Alternatively, the functional changes caused by Munc13-3 loss in the hippocampus may be too subtle to be detectable by the behavioral readouts used in the present study, and/or the hippocampal network might counteract subtle synaptic defects caused by the loss of Munc13-3 in a Munc13-independent homeostatic manner by fine-tuning the functional synaptic connectivity and short-term plasticity.

Future studies will have to focus on fine-tuning the cognition experiments specific to the cerebellum, for instance, by employing a revised Morris water maze task where the focus is placed on self-motion information [11], which depends almost exclusively on the cerebellum. To test the capacity of *Munc13*-*3*-deficient mice to navigate to a hidden platform using self-motion cues, one could test the mice in a modified version of the Morris water maze in darkness, i.e., with infrared light illumination invisible to mice [11]. Similarly, the IntelliCage test could be performed in darkness, thus preventing the mice from using visual cues for spatial orientation and navigation. There is evidence that the dentate gyrus is involved in trace fear conditioning [57] as well as in context discrimination, context generalization, and pattern separation [58–60]. In future studies, it would therefore be interesting to test whether Munc13-3-deficient mice, which lack expression of Munc13-3 in dentate gyrus synapses, are impaired in these dentate gyrus-specific tasks. Finally, in view of the prediction that altered short-term plasticity affects working memory [61], highly stringent working memory tests such as delayed alternation at short time intervals could be used for future analyses of *Munc13*-*3* ^−/−^ mice.

In conclusion, our present study shows that *Munc13*-*3* deficiency alone does not result in an obvious hippocampus-related cognitive deficit but causes a robust reduction in the acoustic startle response, which is detectable in both genders. This readout of a fast cerebellar reflex circuitry obviously requires synaptic vesicle priming by Munc13-3 and Munc13-3-dependent short-term synaptic plasticity for full functionality, thus identifying a unique property of this protein that cannot be compensated for or bypassed.
